# Spectroscopic confirmation of two luminous galaxies at a redshift of 14

**DOI:** 10.1038/s41586-024-07860-9

**Published:** 2024-07-29

**Authors:** Stefano Carniani, Kevin Hainline, Francesco D’Eugenio, Daniel J. Eisenstein, Peter Jakobsen, Joris Witstok, Benjamin D. Johnson, Jacopo Chevallard, Roberto Maiolino, Jakob M. Helton, Chris Willott, Brant Robertson, Stacey Alberts, Santiago Arribas, William M. Baker, Rachana Bhatawdekar, Kristan Boyett, Andrew J. Bunker, Alex J. Cameron, Phillip A. Cargile, Stéphane Charlot, Mirko Curti, Emma Curtis-Lake, Eiichi Egami, Giovanna Giardino, Kate Isaak, Zhiyuan Ji, Gareth C. Jones, Nimisha Kumari, Michael V. Maseda, Eleonora Parlanti, Pablo G. Pérez-González, Tim Rawle, George Rieke, Marcia Rieke, Bruno Rodríguez Del Pino, Aayush Saxena, Jan Scholtz, Renske Smit, Fengwu Sun, Sandro Tacchella, Hannah Übler, Giacomo Venturi, Christina C. Williams, Christopher N. A. Willmer

**Affiliations:** 1 Scuola Normale Superiore, Pisa, Italy; 2 Steward Observatory, University of Arizona, Tucson, AZ USA; 3 Kavli Institute for Cosmology, University of Cambridge, Cambridge, UK; 4 Cavendish Laboratory, University of Cambridge, Cambridge, UK; 5 Center for Astrophysics, Harvard & Smithsonian, Cambridge, MA USA; 6 Cosmic Dawn Center (DAWN), Copenhagen, Denmark; 7 Niels Bohr Institute, University of Copenhagen, Copenhagen, Denmark; 8 Department of Physics, University of Oxford, Oxford, UK; 9 Department of Physics and Astronomy, University College London, London, UK; 10 NRC Herzberg, Victoria, British Columbia Canada; 11 Department of Astronomy and Astrophysics, University of California, Santa Cruz, Santa Cruz, CA USA; 12 Centro de Astrobiología (CAB), CSIC–INTA, Madrid, Spain; 13 European Space Agency (ESA), European Space Astronomy Centre (ESAC), Madrid, Spain; 14 School of Physics, University of Melbourne, Parkville, Victoria Australia; 15 ARC Centre of Excellence for All Sky Astrophysics in 3 Dimensions (ASTRO 3D), Melbourne, Victoria Australia; 16 Institut d’Astrophysique de Paris, Sorbonne Université, CNRS, Paris, France; 17 European Southern Observatory, Garching, Germany; 18 Centre for Astrophysics Research, Department of Physics, Astronomy and Mathematics, University of Hertfordshire, Hatfield, UK; 19 ATG Europe for the European Space Agency, ESTEC, Noordwijk, the Netherlands; 20 European Space Agency, ESTEC, Noordwijk, the Netherlands; 21 AURA for European Space Agency, Space Telescope Science Institute, Baltimore, MD USA; 22 Department of Astronomy, University of Wisconsin-Madison, Madison, WI USA; 23 Astrophysics Research Institute, Liverpool John Moores University, Liverpool, UK; 24 NSF’s National Optical-Infrared Astronomy Research Laboratory, Tucson, AZ USA

**Keywords:** Early universe, Galaxies and clusters

## Abstract

The first observations of the James Webb Space Telescope (JWST) have revolutionized our understanding of the Universe by identifying galaxies at redshift *z* ≈ 13 (refs. ^1–3^). In addition, the discovery of many luminous galaxies at Cosmic Dawn (*z* > 10) has suggested that galaxies developed rapidly, in apparent tension with many standard models^4–8^. However, most of these galaxies lack spectroscopic confirmation, so their distances and properties are uncertain. Here we present JWST Advanced Deep Extragalactic Survey–Near-Infrared Spectrograph spectroscopic confirmation of two luminous galaxies at $z={14.32}_{-0.20}^{+0.08}$ and *z* = 13.90 ± 0.17. The spectra reveal ultraviolet continua with prominent Lyman-α breaks but no detected emission lines. This discovery proves that luminous galaxies were already in place 300 million years after the Big Bang and are more common than what was expected before JWST. The most distant of the two galaxies is unexpectedly luminous and is spatially resolved with a radius of 260 parsecs. Considering also the very steep ultraviolet slope of the second galaxy, we conclude that both are dominated by stellar continuum emission, showing that the excess of luminous galaxies in the early Universe cannot be entirely explained by accretion onto black holes. Galaxy formation models will need to address the existence of such large and luminous galaxies so early in cosmic history.

## Abstract (web summary)

JWST–NIRSpec spectroscopic confirmation of two luminous galaxies is presented, proving that luminous galaxies were already in place 300 million years after the Big Bang and are more common than what was expected before JWST.

## Main

Spectroscopic observations from the James Webb Space Telescope (JWST)–Near-Infrared Spectrograph (NIRSpec)^9^ have been recently carried out for three candidate galaxies at redshift *z* > 14, selected within the JWST Advanced Deep Extragalactic Survey (JADES) campaigns^10,11^. These galaxies were photometrically identified from within the 58 arcmin^2^ observations of the GOODS-S field through JWST observations with up to 13 NIRCam and seven Mid-Infrared Instrument (MIRI) filters^7,12,13^. On the basis of photometry from the Hubble Space Telescope and Cycle 1 JWST–NIRCam data, the probability of these galaxies being low-redshift interlopers was less than 1% (ref. ^12^). By happenstance, the brightest of these three candidate galaxies (hereafter JADES-GS-z14-0) is located at a projected distance of only 0.4 arcsec from a foreground galaxy, and this interloper is at a redshift in which its Balmer break is spectrally coincident with the observed photometric Lyman-α break of the distant galaxy. For this reason, and because of its high inferred luminosity at the photometric redshift, JADES-GS-z14-0 was previously considered a low-redshift interloper with a peculiar spectral energy distribution (SED)^12,13^. The ‘low-redshift solution’ was later disfavoured from the analysis^7^ of the JWST–NIRCam observations carried out in the JADES Origins Field (JOF) programme^11^, which included additional deep medium-band NIRCam observations that substantially strengthened the case for the source being at high redshift.

The three galaxies were observed with NIRSpec in multi-object spectroscopic mode^14^, within a single NIRSpec field of view of 9 arcmin^2^, with both the low-resolution prism and all three medium-resolution gratings probing the wavelength range 0.6–5.2 μm with spectral resolving powers *R* *≈* 100 and *R* *≈* 1,000, respectively. Owing to both the low luminosity of the source and NIRSpec slit losses, the faintest candidate is not significantly detected in the NIRSpec observations (Methods), so hereon we focus on the other two galaxies, JADES-GS-z14-0 and JADES-GS-z14-1, which have been unambiguously detected in the prism spectra.

**Fig. 1:**
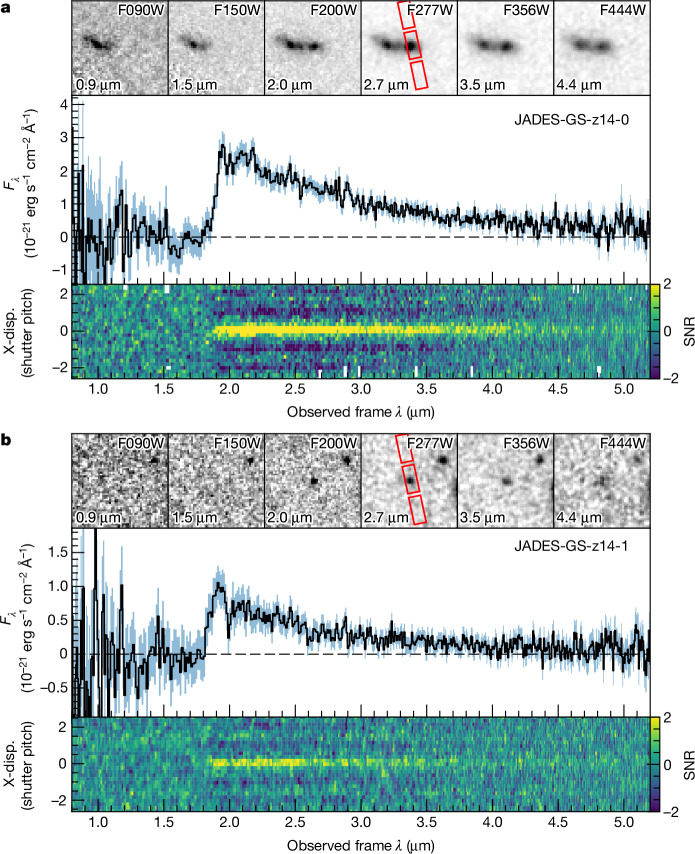
Spectra of the two *z* ≈ 14 galaxies. **a**,**b**, NIRSpec prism (*R* = 100) spectra for JADES-GS-z14-0 (**a**) and JADES-GS-z14-1 (**b**). For each galaxy, the centre panel shows the 1D spectrum (black) and the associated 1*σ* uncertainty (light blue). The bottom panels show the 2D spectrum of the signal-to-noise ratio (SNR) to better highlight the contrast across the break at roughly 1.8 μm. The 2D spectra illustrate the signal-to-noise ratio of the spectra as a function of wavelength and along cross-dispersion direction (X-disp.) Inset stamps in the top panels are cutouts of some of the NIRCam JADES images. The NIRSpec three-shutter slitlets are shown in red in each F277W image.

Figure 1 shows the prism spectra of JADES-GS-z14-0 and JADES-GS-z14-1; there are no prominent emission lines, but both galaxies show a clear break in the flux density with no flux detected blueward of 1.85 μm, the sharpness of which can only be explained as a Lyman-α break^1,2^, placing both galaxies at *z* ≈ 14.

We have also obtained the spectrum of the low-redshift galaxy 0.4 arcsec east of JADES-GS-z14-0, which has revealed several prominent emission lines (for example, [OIII]*λλ*4959,5007 and Hα), placing this projected nearby source at a redshift of *z* = 3.475 (Methods). At this redshift, its Balmer break is at 1.62 μm, excluding the possibility that the sharp break in the flux density at 1.85 μm observed in the spectrum of JADES-GS-z14-0 is caused by contamination from the nearby foreground source. The presence of the nearby low-redshift galaxy, however, mildly boosts the luminosity of JADES-GS-z14-0 by means of gravitational lensing. We have verified that the magnification factor is less than a factor of 1.2 (Methods).

A redshift determination for galaxies within the epoch of reionization based solely on the Lyman-α break is sensitive to the absorption of neutral hydrogen along the line of sight^1,3,15^. We have thus estimated the redshift of the two galaxies by parameterizing the rest-frame UV continuum emission with a power law of the form ${F}_{\lambda }\propto {\lambda }^{\beta }$ and taking into account the many physical processes that can shape the Lyman-break profile in the prism spectra (Methods). The redshifts we have recovered from our best-fitting models are $z={14.32}_{-0.20}^{+0.08}$ and *z* = 13.90 ± 0.17 for JADES-GS-z14-0 and JADES-GS-z14-1, respectively.

In the redshift range inferred by fitting the Lyman-break profile, we have also found a tentative detection of CIII]*λλ*1907,1909 (hereafter CIII]) emission at 2.89 μm in JADES-GS-z14-0 (Methods) at a level of significance of 3.6*σ*. If confirmed in future NIRSpec observations, this line yields a redshift of 14.178 ± 0.013 and the presence of damped Lyman-α absorption with a neutral hydrogen column density of ${\log }_{10}\left({N}_{{\rm{HI}}}/{\rm{c}}{{\rm{m}}}^{-2}\right)=22.23\pm 0.08$ is necessary to match the wavelength and shape of the Lyman-α break.

**Fig. 2:**
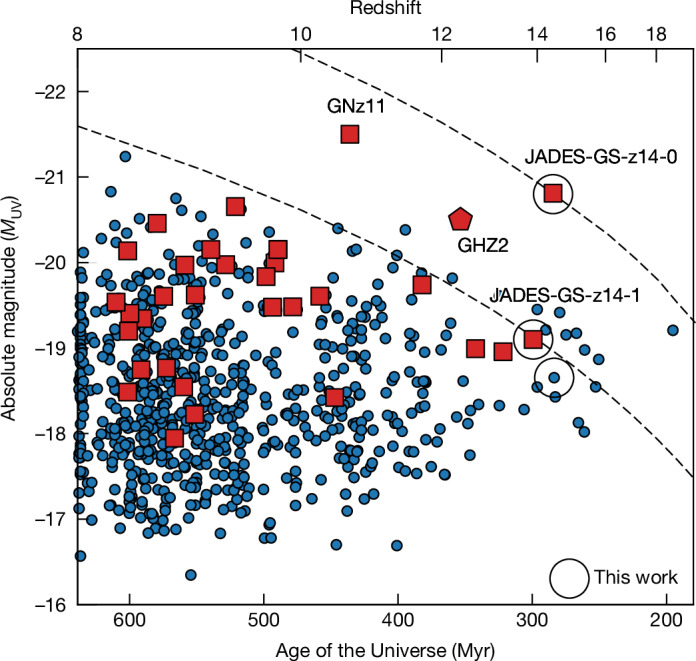
UV absolute magnitudes of galaxies at *z* > 8. The blue circles are candidate high-*z* galaxies in the GOODS-S and GOODS-N identified in JADES^12^ and the red squares are the spectroscopically confirmed galaxies^19,44,45^. For comparison, we also report the galaxy GHZ2 (refs. ^16,17^) from the Grism Lens-Amplified Survey from Space (red pentagon). The empty black circles highlight the targets analysed in this work. The relatively low number of galaxies near *z* = 10 is an artefact of photometric-redshift selections. The dashed lines illustrate a semi-empirical luminosity evolution ($\propto {(1+z)}^{-4.5}$) of haloes of a given comoving abundances.

These are the earliest galaxies with spectroscopically confirmed redshifts, exceeding the previous high marks of *z* = 13.2 (refs. ^1,3^) and *z* = 13.07 (ref. ^2^). Furthermore, these two galaxies are luminous with a rest-frame UV absolute luminosity at 1,500 Å of *M*_UV_ = −20.81 and *M*_UV_ = −19.00, respectively. We particularly highlight JADES-GS-z14-0, which despite its redshift is the third most UV luminous of the 700 *z* > 8 candidates in JADES, twice more luminous than GHZ2 (refs. ^16,17^), and only a factor of two less luminous than GN-z11 (refs. ^18,19^). We illustrate the distribution of UV luminosity and redshift in Fig. 2. We stress that the high luminosity is particularly important in view of the rapidly evolving halo mass function expected in cold-dark-matter cosmology. From an *N*-body simulation run with Abacus^20^, we estimate that the halo mass threshold required to yield a fixed comoving abundance varies as ${(1+z)}^{-6}$ for this region of mass and redshift. A dimensional scaling for luminosity would be halo mass divided by the age of the Universe, which is scaling as ${(1+z)}^{-3/2}$, yielding a simple baseline that luminosities might scale as ${(1+z)}^{-4.5}$. Overplotting such a scaling on Fig. 2 shows how remarkable JADES-GS-z14-0 is: it shows most notably that some astrophysical processes are creating a deviation from the dimensional scaling of halo mass and the Hubble time. Even JADES-GS-z14-1, although more similar in *M*_UV_ to the lower redshift family, is distinctively luminous by this metric. We, therefore, argue that these two galaxies, and particularly JADES-GS-z14-0, provide a crisp spectroscopic confirmation to the trend that has been inferred several times from photometric samples^4,6–8^ that the galaxy UV luminosity function evolves slowly, with more luminous galaxies at high redshift than predicted in a variety of pre-JWST predictions. Having established the remarkable redshifts and luminosities of these sources, we now turn to a more detailed analysis of them.

From the spectrum redward of the break, we measure a power-law index *β*, also known as the UV slope, of −2.20 ± 0.07 and −2.71 ± 0.19 for JADES-GS-z14-0 and JADES-GS-z14-1, respectively. These results indicate that the emission is dominated by a relatively young (less than 300 Myr) stellar population and low dust attenuation^21–23^. We note that the stellar UV slope could be also modified by two-photon and free-bound nebular continuum emission^21–27^. However, we can rule out a strong two-photon contribution in our galaxies due to the lack of the characteristic peak at 1,500 Å (ref. ^27^). The absence of emission lines disfavours free-bound emission, but this possibility cannot be fully ruled out because, at *z* ≈ 14, NIRSpec does not cover the Balmer break nor any Balmer emission lines.

The physical properties of the two galaxies have been inferred by means of spectro-photometric modelling of their SEDs within a Bayesian framework. The details of the modelling and the posterior distribution of free parameters are discussed in Methods, whereas the galaxy properties are reported in Table 1. The inferred star-formation history indicates that these galaxies have grown their masses over the last 100 Myr, suggesting that the observed stellar population started forming at *z* ≈ 20 with a rapid growth up to *z* ≈ 14 (ref. ^28^). We also note that the SED modelling favours a high escape fraction of ionizing photons (${f}_{\mathrm{esc}}^{\mathrm{LyC}} > 0.35$) to reproduce the blue UV slopes and the absence of emission lines in both galaxies.

**Table 1:** Galaxy properties

**ID**	**JADES-GS-z14-0**	**JADES-GS-z14-1**
Extended ID	JADES-GS-53.08294−27.85563	JADES-GS-53.07427−27.88592
NIRCam ID	183348	18044
Right ascension (ICRS)	3 h 32 min 19.905 s	3 h 32 min 17.825 s
Declination (ICRS)	−27° 51′ 20.27″	−27° 53′ 09.33″
Redshift	${14.32}_{-0.20}^{+0.08}$	13.90 ± 0.17
UV slope *β*	−2.20 ± 0.07	−2.71 ± 0.19
*M*_UV_	−20.81 ± 0.16^b^	−19.0 ± 0.4
UV radius (*r*_UV_) (pc)	260 ± 20	<160
log_10_(*M*_star_/*M*_☉_)^a^	${8.6}_{-0.2}^{+0.7}$^b^	${8.0}_{-0.3}^{+0.4}$
${{\rm{S}}{\rm{F}}{\rm{R}}}_{100}\,({M}_{\odot }\,{{\rm{y}}{\rm{r}}}^{-1})$	${4}_{-3}^{+9}$^b^	${1.2}_{-0.9}^{+0.7}$
${{\rm{S}}{\rm{F}}{\rm{R}}}_{10}\,({M}_{\odot }\,{{\rm{y}}{\rm{r}}}^{-1})$	19 ± 6^b^	${2}_{-0.4}^{+0.7}$
${{\rm{sSFR}}}_{10}\,({{\rm{Gyr}}}^{-1})$	${45}_{-35}^{+56}$	${18}_{-38}^{+75}$
*A*_V_ (mag)	${0.31}_{-0.07}^{+0.14}$	${0.20}_{-0.07}^{+0.11}$
${\log }_{10}\left(Z/{Z}_{\odot }\right)$	$-{1.5}_{-0.4}^{+0.7}$	$-{1.1}_{-0.5}^{+0.6}$
${f}_{{\rm{esc}}}^{{\rm{LyC}}}$	${0.84}_{-0.16}^{+0.09}$	${0.63}_{-0.29}^{+0.25}$

Galaxy properties inferred from NIRSpec data corrected for slit losses based on NIRCam fluxes. ^a^Uncertainties refer only to the internal statistical errors of our model. Stellar mass is sensitive to the assumptions on star-formation history^28^. ^b^Corrected for gravitational lensing amplification of *μ* = 1.17 (Methods).

**Fig. 3:**
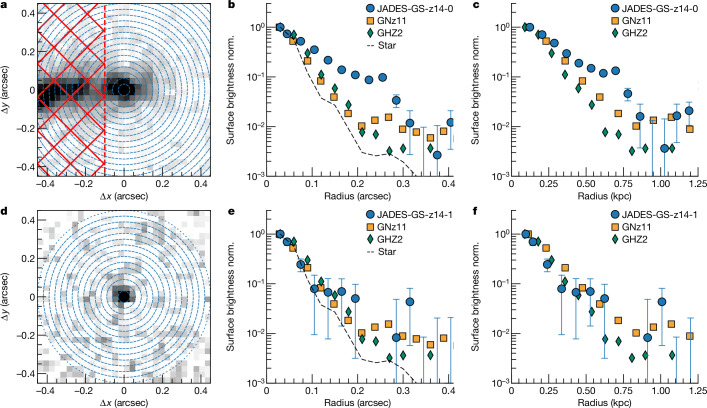
Galaxy size. **a**–**f**, The normalized (norm.) radial profiles of the observed surface brightness at 2 μm of JADES-GS-z14-0 (**a**–**c**) and JADES-GS-z14-1 (**d**–**f**). From left to right, the panels show the NIRCam images in the F200W filter (**a**,**d**), the surface brightness profiles in arcsec units (**b**,**e**) and the surface brightness profiles in kpc units (**c**,**f**). The circularized light profiles are extracted from the rings marked with blue concentric circles in the NIRCam image, and the red dashed region in the JADES-GS-z14-0 image marks the part that was masked to remove the contamination of the neighbouring *z* = 3.475 galaxy. The comparison with the radial profile of a star in the field of view (grey dashed curve), GN-z11 (ref. ^46^) (orange squares) and GHZ2 (ref. ^16^) (green diamonds) shows that JADES-GS-z14-0 is significantly more extended than the most luminous galaxies previously known at *z* > 10. The error bars in **b**, **c**, **e** and **f** show the 1*σ* uncertainties of the surface brightness measurements.

The NIRCam images of JADES-GS-z14-0 clearly show that the source is extended, whereas JADES-GS-z14-1 is more compact. Figure 3 shows the radial profile of the emission at 2 μm of the two galaxies. The radial surface brightness profile of JADES-GS-z14-0 shows emission extended up to 1 kpc (kilopc), significantly beyond the point spread function of JWST. We also note that the profile is significantly more extended than the UV emission of the two more luminous galaxies at *z* > 10: GN-z11 (refs. ^19,29^) and GHZ2 (refs. ^16,17^). Using ForcePho (Methods) to fit the imaging data, we find that the galaxy is well fit by an elliptical exponential profile with a deconvolved half-light radius (*r*_UV_) of 0.079 ± 0.006 arcsec and 260 ± 20 pc. This large size indicates that the UV light of JADES-GS-z14-0 is produced mainly by a spatially extended stellar population, excluding a dominant contribution by an active galactic nucleus (AGN). This differs from other more compact high-luminosity galaxies, in which some studies have suggested that an unobscured AGN is dominating the UV light^29,30^.

The rest-frame UV emission of JADES-GS-z14-1 seems compact and marginally resolved by the NIRCam point spread function. The forward modelling of the light profile returns an upper limit on *r*_UV_ < 160 pc, which agrees with the compact size determined for other low-luminosity *z* > 10 galaxies^3,7,31^. For this galaxy, the morphological analysis is not sufficient to exclude the presence of a luminous AGN, but the inferred UV slope of −2.71 ± 0.19 suggests that the light is mainly emitted by stars in the galaxy. The slope expected for the emission of an AGN accretion disc is, on average, of the order of −2.3 or shallower^32,33^, and there are no standard mechanisms that are able to reproduce a steeper profile without invoking a strong contribution from the emission of a young (less than 50 Myr) stellar population^21–23^.

The best-fitting SED models presented in Table 1 indicate a modest but non-zero amount of reddening by dust, with an *A*_V_ of 0.2–0.3 mag for both galaxies. These results are in agreement with recent models proposed to explain the presence of luminous galaxies such as JADES-GS-z14-0 and JADES-GS-z14-1 at early times^34,35^. Such models expect that *z* > 10 galaxies have lower dust content in the interstellar medium than equal-mass galaxies at lower redshifts, despite the rapid (roughly 10 Myr) dust enrichment from supernovae^36^. Indeed, if our massive *z* ≈ 14 galaxies had a stellar-to-dust ratio of about 0.002, similar to those observed in *z* ≈ 6 galaxies^37^, the dust attenuation would be a factor at least four times higher (*A*_V_ > 1 mag), due to their compact UV size, than what is observed^34^. The moderate dust attenuation in our galaxies can be explained by different scenarios: (1) a large amount of dust distributed on large scales due to galactic outflows, reducing the observed dust attenuation^34,38^; (2) a different dust composition^39^ and so dust mass absorption coefficient and (3) a high destruction rate of dust grains due to shock waves generated by supernovae explosions^36^. Independently from the proposed scenarios, our observations indicate that the properties of galaxies seem to change rapidly in only 600 Myr (that is, from *z* = 14 to *z* = 6).

In conclusion, the presented spectroscopic observations of JADES-GS-z14-0 and JADES-GS-z14-1 confirm that bright and massive galaxies existed already only 300 Myr after the Big Bang, and their number density^7^ is more than ten times higher than extrapolations based on pre-JWST observations^40^. The morphology and UV slope analysis help rule out a significant AGN contribution for either galaxy. Other potential explanations, such as dust content^34^, star-formation processes^8,41^ and a top-heavy initial mass function^42,43^, must be investigated to explain the excess of luminous galaxies in the early Universe.

In the context of future observations, we stress that JADES-GS-z14-0 is unexpectedly and remarkably luminous. The spectroscopic confirmation of this source indicates the existence of many similar galaxies, particularly when considering the relatively small survey area of JADES. Galaxies such as this are sufficiently luminous for follow-up observations with the Atacama Large Millimeter/Submillimeter Array and MIRI, promising to open the view to the rest-frame optical and far-infrared at Cosmic Dawn, the period during which the first galaxies were born.

## Methods

### Cosmology model and definitions

Throughout this study, we adopt the standard lambda cold dark matter cosmology model and assume the following cosmological parameters: Hubble constant *H*_0_ = 70 km s^−1^ Mpc^−1^, matter density parameter *Ω*_m_ = 0.3 and cosmological constant *Ω*_*Λ*_ = 0.7. One arcsec at *z* = 14 corresponds to a physical scale of 3.268 kpc. All magnitudes are presented in the AB magnitude system, and the term SFR_10_ refers to the star-formation rate (SFR) averaged over the past 10 Myr. Equivalent widths of emission lines are quoted in the rest frame. The absolute UV magnitude is estimated at the rest-frame wavelength of 1,500 Å.

### NIRSpec observations and data reductions

The NIRSpec data used in this work are part of the Guaranteed Time Observations programme ID 1287. The micro-shutter array (MSA) mask was designed with the eMPT software^47^ and proceeded using the same method as described in refs. ^19^,^45^. The NIRSpec pointing was optimized for six out of seven of the highest priority targets in the catalogue, which include the galaxies analysed in this work. The eMPT software guarantees that all galaxies are located within roughly 90 milliarcseconds (mas) from the centre of the shutter in the dispersion direction and within roughly 220 mas in the spatial direction. We note that given the number of targets, not all highest priority galaxies are placed at the centre on the shutter where the slit loss effect is minimal.

The observations were carried out between 10 and 12 January 2024. Three consecutive visits were scheduled for the programme, but the visit 2 was not performed because of a loss of lock on the guide star. The two obtained visits, 1 and 3, differed in their pointing by less than 1 arcsec but they have the same position angle. The MSA configurations were designed to place the highest priority targets in the same position within the shutter in all visits. JADES-GS-z14-1 was observed in both visits, whereas JADES-GS-z14-0 was observed only in the visit 1, as the visit 3 was set up to observe the nearby low-redshift galaxy to exclude contamination.

The disperser–filter configurations used in the programme were PRISM–CLEAR, G140M–F070LP, G235M–F170LP, G395M–F290LP and G395H–F290LP. The first four spectral configurations provided spectroscopic data with spectral resolving power of *R* = Δ*λ*/*λ* ≈ 100 and *R* ≈ 1,000 in the wavelength range between 0.6 and 5.2 μm. The G395H–F290LP disperser–filter configuration covered the wavelength range 2.87–5.27 μm with a spectral resolving power of *R* ≈ 2,700.

For the PRISM–CLEAR configuration, four sequences of three nodded exposures were used for each pointing, whereas one sequence of three nodded exposures was used for the spectral configuration of the gratings. Each nodded exposure sequence consisted of six integrations of 19 groups in NRSIRS2 readout mode^48^, resulting in an exposure time of 8,403.2 s. The total exposure times for each target are reported in Extended Data Table 1.

We made use of the NIRSpec guaranteed time observations (GTO) pipeline (S.C. et al., manuscript in preparation) to process the data. The pipeline was developed by the European Space Agency NIRSpec Science Operations Team and the NIRSpec GTO Team. A general overview of the data processing is reported in refs. ^19^,^45^. To optimize the signal-to-noise ratio of the data, we used the 1D spectra extracted from an aperture of 3 pixels, corresponding to 0.3 arcsec, located at the target position in the 2D spectra.

**Extended Data Fig. 1:**
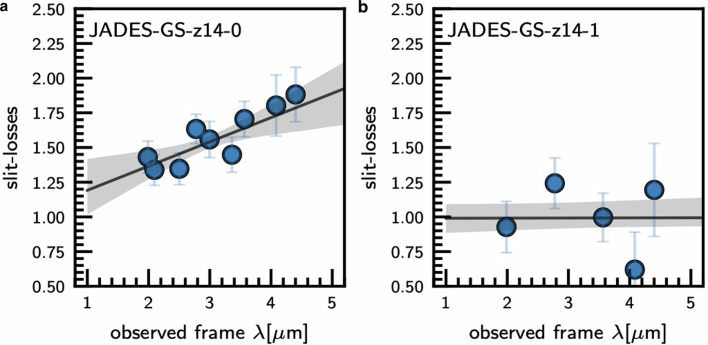
NIRSpec slit-losses. **a-b**, Slit-losses as a function of wavelength for JADES-GS-z14-0 (**a**) and JADES-GS-z14-1 (**b**). Slit-losses are estimated by comparing the NIRSpec continuum level and NIRCam fluxes. The first-order polynomial best fits and uncertainties are reported as black lines and gray-shaded regions.

The pipeline applies a wavelength-dependent slit-loss correction to the measured flux, based on the position of the source inside the micro-shutter and assuming a point-source geometry. To verify the quality of this correction, we compare the pipeline-corrected fluxes to the NIRCam photometric measurements. For JADES-GS-z14-0, which is spatially extended, we used the NIRCam measurements derived with ForcePho (section ‘Morphological analysis’). We found that roughly 30% (roughly 50%) of the total flux is lost at 2(5) μm in the NIRSpec data (Extended Data Fig. 1). We used a first-order polynomial *α*_1_ + *α*_0_*λ* to fit the slit losses as a function of wavelength (*λ*) and found *α*_0_ = 0.18 ± 0.11 m^−1^ and *α*_1_ = 1.0 ± 0.2.

For JADES-GS-z14-1, the NIRCam fluxes inferred from an aperture of 0.2 arcsec are consistent with the NIRSpec spectrum, indicating that the slit-loss correction applied by the GTO pipeline is sufficient to recover the total light of this compact source (Extended Data Fig. 1). In this case, we estimated *α*_0_ = 0.001 ± 0.025 μm^−1^ and *α*_1_ = 0.98 ± 0.12.

### Imaging data

Photometry for the candidate *z* ≈ 14 galaxies studied in this work was taken from JWST–NIRCam imaging catalogues of JADES^10^, with supplemental imaging data from the First Reionization Epoch Spectroscopic Complete Survey^49^ and JOF^50^ programmes. These data were reduced together following the procedure outlined in ref. ^11^. The resulting mosaics include both observations taken in late 2022 as well as additional JADES observations taken in late 2023, and reach 5*σ* observational depths of 2.4 nJy in F200W using a 0.2″ diameter aperture. We present NIRCam thumbnails centred on JADES-GS-z14-0 and JADES-GS-z14-1 in the top panels of Fig. 1.

The sources were initially selected from the modelling of the photometry presented in ref. ^12^. Two of the sources we explore, JADES-GS-z14-1 (JADES-GS-53.07427-27.88592 in ref. ^12^), and the faintest galaxy described herein (JADES-GS-53.10763−27.86014) were part of the primary sample of *z* > 8 galaxies in ref. ^12^, with photometric redshifts of ${z}_{{\rm{phot}}}={14.36}_{-1.4}^{+0.82}$ and ${z}_{{\rm{phot}}}={14.44}_{-1.2}^{+0.97}$, respectively. JADES-GS-z14-0 (JADES-GS-53.08294−27.85563) was presented in ref. ^12^ (at ${z}_{{\rm{phot}}}={14.51}_{-0.28}^{+0.27}$) but initially rejected in that study due to the morphology, brightness and the proximity of the source to the neighbouring galaxy with photometric evidence of a Balmer break at roughly 1.7 μm.

In October 2023, JADES-GS-z14-0 and JADES-GS-53.10763−27.86014 were also observed as part of the JOF programme^50^, which included a NIRCam pointing of 104 h of total exposure spread between six medium-band filters (F162M, F182M, F210M, F250M, F300M, F335M). These filters were chosen to help refine high-redshift galaxy selection in this ultra-deep region of the JADES footprint. NIRCam medium bands can be used to trace the galaxy stellar continuum and aid in rejecting sources at low redshift with strong emission lines that have similar wide-filter colours to high-redshift galaxies^51–53^. In ref. ^7^, the authors used JOF photometry to select a sample of nine candidate galaxies at *z* = 11.5 − 15, including JADES-GS-z14-0. The additional medium-band observations for this source had a best-fit photometric redshift of ${z}_{{\rm{phot}}}={14.39}_{-0.09}^{+0.23}$, and fits at *z* < 7 were effectively ruled out because of the lack of flux observed shortward of the Lyman-α break, the strength of the break indicated by the F182M–F210M colour and the F250M flux tracing the UV continuum. The authors also estimated a UV slope of −2.40 ± 0.12 and a size of 260 ± 6 pc, which are consistent with those inferred in this study. Robertson et al.^7^ also presented the evolution of the UV luminosity function and cosmic SFR density at *z* > 14 inferred from observations of JADES-GS-z14-0, and we refer the reader there for more details.

For this analysis, we fit JADES-GS-z14-0 using ForcePho (B.D.J.o et al., manuscript in preparation) to properly disentangle the flux of this source from the neighbour (Extended Data Table 2). For JADES-GS-z14-1, as this source was isolated and much more compact, we extracted fluxes using aperture photometry with an 0.2″ aperture (Extended Data Table 2), and applied an aperture correction assuming a point source. In a companion paper^28^, our team presents JWST–MIRI photometry of JADES-GS-z14-0 from ultra-deep 43 h F770W imaging from programme ID 1180. This measures F770W to be 74 ± 5 nJy, mildly above the 3–5 μm photometry of roughly 47 nJy, probably due to the presence of strong emission lines in F770W. Helton et al.^28^ also discuss the implications of this rest-optical finding.

### The third candidate JADES-GS-53.10763−27.86014

**Extended Data Fig. 2:**
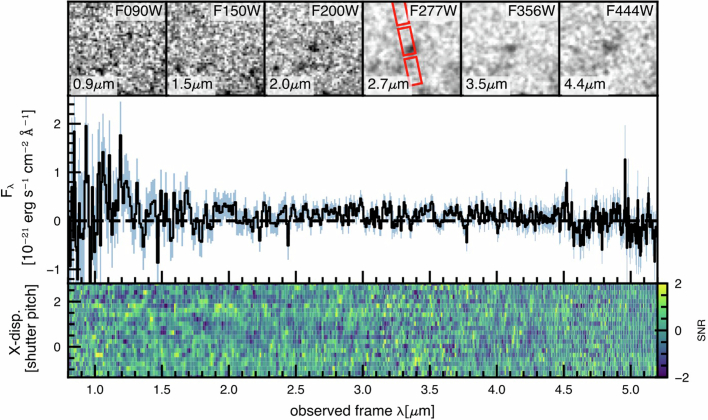
NIRSpec spectrum of the third z~14 candidate galaxy. 1D (middle) and 2D (bottom) PRISM/CLEAR spectra of JADES-GS-53.10763-27.86014, the third candidate $z \sim 14$ galaxy observed with NIRSpec in the observing program 1287. Due to the low signal-to-noise ratio, no redshift can be inferred from this spectrum. The top row shows cutouts of NIRCam F090W, F150W, F200W, F277W, F356W, and F444W images. NIRSpec micro-shutter 3-slitlet array aperture is reported in red in the F277W image.

The candidate *z* ≈ 14 galaxy JADES-GS-53.10763-27.86014 identified by Hainline et al.^12^ and Robertson et al.^7^. The NIRCam images show a clear dropout in bluer filters, yielding a photometric redshift of ${z}_{{\rm{phot}}}={14.63}_{-0.75}^{+0.06}$ (ref. ^7^). The target was observed in visits 1 and 3 of the NIRSpec programme by using the same shutter position in both visits. Extended Data Fig. 2 illustrates the 1D and 2D spectra of the galaxy. Only a faint continuum emission is barely detected in the NIRSpec spectrum with a significance level less than 1*σ*. The signal is not sufficient to confirm or rule out the photometric redshift determined by NIRCam images. We believe that the NIRSpec slit losses contribute to the low signal-to-noise ratio of the data. The target is located at the edge of the shutter and, despite its compact size, we expect that about 20% of the light is lost at 2 μm and 35% at 5 μm. The slit losses are two times higher than those of JADES-GS-z14-1, which is also 1.6 times more luminous than JADES-GS-53.10763−27.86014. In conclusion, the low sensitivity of these observations does not allow us to confirm or rule out the photometric redshift for this target.

### The low-redshift galaxy close to JADES-GS-z14-0

**Extended Data Fig. 3:**
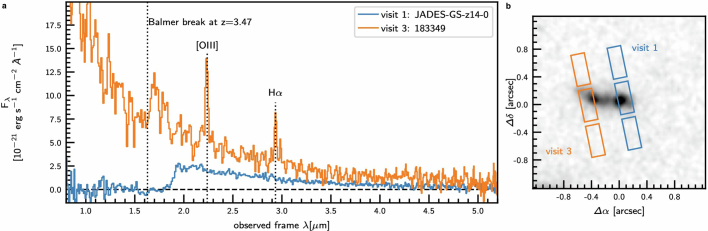
Low-redshift neighbouring galaxy. **a**, Spectra of JADES-GS-z14-0 (blue) and of the target 183349 (orange). This latter is within 0.4 arcsec from JADES-GS-z14-0, as shown in the NIRCam F277W cutout in the right panel (**b**). The MSA slitlets used for the two targets are overplotted in blue and orange, respectively.

The identification of the neighbouring galaxy with NIRCam ID 183349 at 0.4 arcsec from JADES-GS-z14-0 initially raised several doubts about the photometric redshift of the high-redshift target as the potential Lyman-α break for this object could be a Balmer break if these two sources are associated at similar redshifts. Therefore, we dedicated the visit 3 of the NIRSpec programme 1287 to observe the neighbouring galaxy and assess any possible contamination and constrain the gravitational lensing effect. Extended Data Fig. 3 shows the spectrum of the target 183349. The doublet [OIII]*λλ*4959,5007 and Hα emission lines are detected with a high level of significance in both prism and grating spectra, yielding a secure spectroscopic redshift of *z* = 3.475 (in agreement with the photometric redshift *z*_phot_ = 3.4 ± 0.2 from ref. ^12^). The spectrum also reveals a clear Balmer break feature at roughly 1.6 μm. Therefore, we can rule out the drop at roughly 1.9 μm observed in JADES-GS-z14-0 being due to the contamination of the neighbouring galaxy. Finally, 183349 has no bright emission lines at observed wavelengths at 2.89 μm, where we detect tentative CIII] emission in JADES-GS-z14-0. We can thus rule out that the tentative CIII] is due to contamination from 183349.

**Extended Data Fig. 4:**
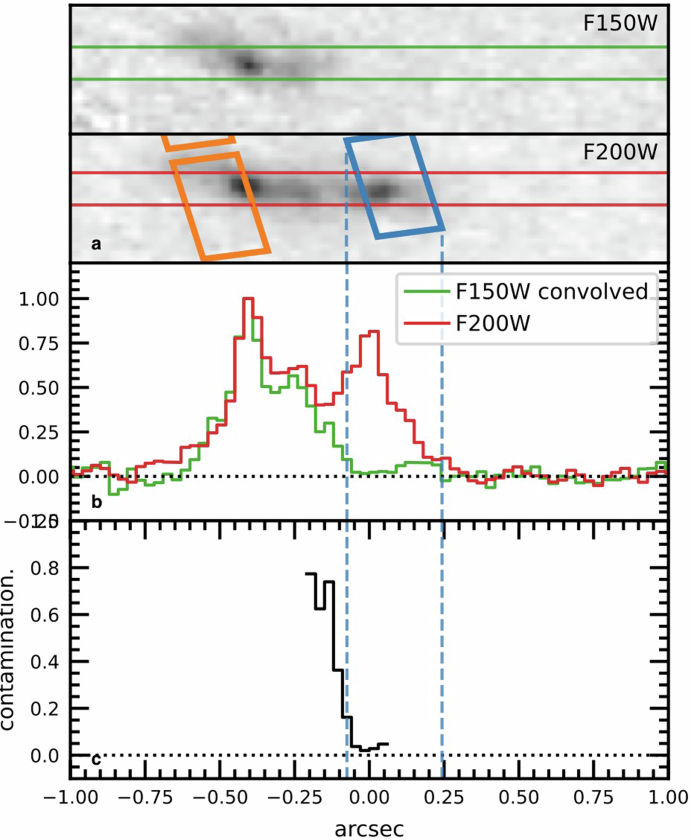
Light profile of the foreground galaxy. **a–b**, F150W (**a**) and F200W (**b**) NIRCam images of JADES-GS-z14-0. The NIRSpec micro-shutter slits of visits 1 and 3 are illustrated in blue and orange, respectively. F150W image is smoothed to the same angular resolution as F200W NIRCam image. The green and red lines show the box-car extraction regions from which we obtained the light profiles reported with the same colors in the third panel. The profiles are normalized to the peak of the foreground galaxy. **c**, Ratio between the F200W and F150W light profiles in the region where the two galaxies spatially overlap. This ratio quantifies the contamination of the foreground galaxy to JADES-GS-z14-0. Vertical blue dashed line shows the projection of the NIRSpec micro-shutter slit into the radial profile.

As the foreground galaxy might contaminate the spectrum of the JADES-GS-z14-0, we analyse the surface brightness profile of the two galaxies. Extended Data Fig. 4 shows the light profiles from the F150W and F200W NIRCam images extracted from a slit-oriented east to west and as large as 0.15 arcsec so that the slit includes both galaxies. JADES-GS-z14-0 is absent in the F150W NIRCam image, and thus, we used the F150W profile to quantify the contamination. Before the extraction, F150W NIRCam was smoothed to the same angular resolution of F200W data. Extended Data Fig. 4 reports the light profiles in the two filters normalized to the peak at the location of the foreground source (that is, roughly 0.4 arcsec from the JADES-GS-z14-0). Assuming that the surface brightness profile of the foreground galaxy in the F200W image is similar to that at F150W wavelengths, we estimated a contamination of less than 10% at the location of JADES-GS-z14-0. The last panel of Extended Data Fig. 4 indeed illustrates the ratio between the light profile in F200W filter and that in F150W from −0.2 arcsec (that is, −0.6255 kpc) to 0 arcsec with respect to the centre of JADES-GS-z14-0. This spatial range corresponds to the region in which the light of the two galaxies might overlap. The contamination is of the order of 70% at 650 pc from the centre of JADES-GS-z14-0 and drops rapidly to less than 20% at 350 pc from the galaxy. As the top-left edge of the NIRSpec shutter is located at −0.08 arcsec from the centre of JADES-GS-z14-0, we concluded that the contamination of the light of the foreground galaxy is negligible in the NIRSpec spectrum. Therefore, the contamination of the low-*z* galaxy on the NIRSpec spectrum is lower than 10%.

We have also verified that the magnification provided by the foreground galaxies to JADES-GS-z14-0 is limited (*μ* < 1.2). We use the software lenstool^54^ to construct lens model of ID 183349 and another galaxy, JADES-GS-53.08324-27.85619 (ID 182698; *z*_phot_ ≈ 2.04) that is 2.2 arcsec from JADES-GS-z14-0. On the basis of Hubble Space Telescope/ACS and JWST–NIRCam SED, we infer stellar mass $\log ({M}_{{\rm{s}}{\rm{t}}{\rm{a}}{\rm{r}}}/{M}_{\odot })=8.7\pm 0.1$ and 9.7 ± 0.1 for ID 183349 and 182698, respectively. We then derived integrated velocity dispersions of 53 and 100 km s^−1^ assuming the stellar-mass Tully–Fisher relation measured at *z* ≈ 2.3 (ref. ^55^). Assuming a singular isothermal spherical distribution of matter in these two foreground potentials, we derive a modest lensing magnification factor of *μ* = 1.17 at the location of JADES-GS-z14-0. Such a magnification factor is corrected for when we derived the physical properties: that is, luminosities and masses.

### Redshift determination

The photometric redshifts of JADES-GS-z14-0 and JADES-GS-z14-1 are ${z}_{a}={14.39}_{-0.09}^{+0.23}$ (ref. ^7^) and ${z}_{{\rm{phot}}}={14.36}_{-1.4}^{+0.82}$ (ref. ^12^), respectively. The strong Lyman breaks (flux ratio between 1.90–2.1 and 1.5–1.8 μm higher than 9) observed by NIRSpec in both JADES-GS-z14-0 and JADES-GS-z14-1 confirm both galaxies to be at a redshift of about 14.

Recent studies have shown that the profile of the Lyα spectral break does not depend only on intergalactic medium absorption but can also be modulated by: (1) neutral gas in the galaxy or in the surrounding medium^15,56,57^; (2) the presence of a local ionized bubble^58,59^; (3) Lyα line emission that would enhance the flux of spectral channels containing the line in the low-resolution data^60^. Therefore, to determine the spectroscopic redshift, we model the continuum emission with a power-law function (${f}_{\lambda }\propto {\lambda }^{\beta }$), which reproduces well the rest-frame UV continuum emission in galaxies with young stellar populations^21–23,61^, and absorption of neutral hydrogen following the prescriptions discussed in refs. ^59^ and ^3^. The intergalactic medium transmission is modelled following Mason and Gronke^62^ and depends on two free parameters: the global neutral gas fraction (*x*_HI_) and the ionized bubble size (*R*_ion_). We assumed a flat prior for the neutral gas fraction over the range ${x}_{{\rm{HI}}}\in \left[{\rm{0.95,1}}\right]$ and a flat prior distribution for the ionized bubble size over the range ${R}_{{\rm{ion}}}\in \left[{\rm{0.1,1}}\right]$ proper Mpc. These are the expected values for a typical galaxy with a *M*_UV_ ≈ −20 at *z* = 14 (ref. ^63^). As the Lyman-α drop profile can also be caused by dense neutral gas in the circumgalactic medium and located along the line of sight (that is, damped Lyman-α absorption) following refs. ^15^ and ^3^, we parametrized this additional absorption by the column density of neutral hydrogen, $\log \left({N}_{{\rm{HI}}}/{{\rm{cm}}}^{-2}\right)$, and assumed a flat prior $\log \left({N}_{{\rm{HI}}}/{{\rm{cm}}}^{-2}\right)\in \left[{\rm{10,28}}\right]$. Finally, recent studies^60,64^ have also shown that the Lyα emission line can modify the prism spectra and so alter the redshift measurement. Therefore, we added a mock spectroscopically unresolved emission line in our model to represent the Lyα emission. The line model was parametrized by the rest-frame equivalent width ($\log \left(E{W}_{0}/{\text{\AA }}\right)\in \left[-\mathrm{2,2}\right]$) and velocity shift with respect to systemic ($\varDelta v\in \left[0,\,\mathrm{3,000}\right]{\rm{km}}\,{{\rm{s}}}^{-1}$). This latter mimics the effects of outflows and resonant scattering on Lyα line emission^65^.

**Extended Data Fig. 5:**
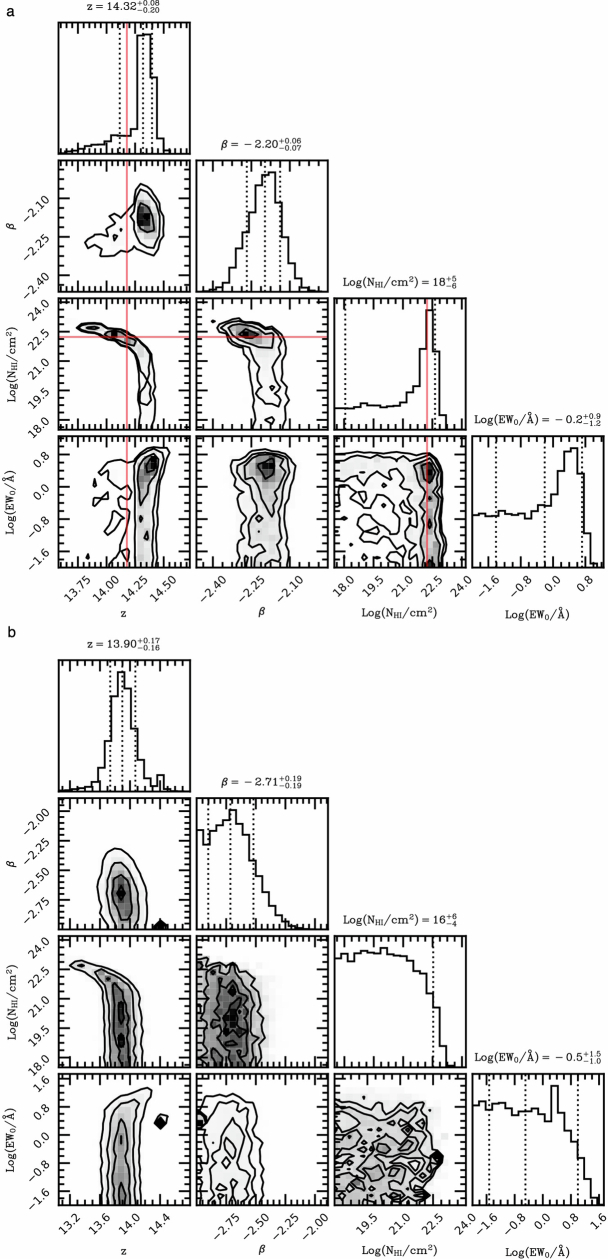
Redshift determination. **a–b**, Results of the spectral fitting of JADES-GS-z14-0 (**a**) and JADES-GS-z14-1 (**b**) while varying the redshift ($z$), UV slope (${f}_{\lambda }\propto {\lambda }^{\beta }$), fraction of neutral hydrogen in the IGM (${x}_{{HI}}$), hydrogen column density of the additional damped Lyman-${\rm{\alpha }}$ absorption (log$\left({N}_{{HI}}/c{m}^{-2}\right)$), size of the ionized bubble (${R}_{{ion}}$), equivalent width (log$\left(E{W}_{0}/{\text{\AA }}\right)$) and velocity shift ($\varDelta v$) of Lyman-${\rm{\alpha }}$ line. The corner plots show the posterior distribution functions and the covariance between the parameters. The posterior distribution of ${x}_{{HI}}$, ${R}_{{ion}}$, and $\varDelta v$ is not illustrated because they are flat. The dotted lines indicate 16th, 50th, and 84th percentile of the marginalized posterior distributions. The red lines in the corner plot of JADES-GS-z14-0 represent the estimates of redshift and log$\left({N}_{{HI}}/c{m}^{-2}\right)$ based on the potential CIII]$\lambda 1909$ emission line.

Extended Data Fig. 5 shows the posterior distributions of the free parameters used to fit the data of JADES-GS-z14-0 and JADES-GS-z14-1. The posteriors of the parameters *x*_HI_, *R*_ion_ and *Δv* are flat for both galaxies and are not reported in the corner plot. The best-fit redshifts are ${14.32}_{-0.20}^{+0.08}$ and 13.90 ± 0.17, respectively, for the two targets. The profile of the posterior distributions of $\log \left({N}_{{\rm{HI}}}/{{\rm{cm}}}^{-2}\right)$ exclude the presence of dense damped Lyman-α absorption with $\log \left({N}_{{\rm{HI}}}/{{\rm{cm}}}^{-2}\right) > 23.64$, but does not preclude less dense absorbing systems along the line of sight. The results also indicate that the rest-frame equivalent width of the Lyman-α line is lower than 10 Å.

### Emission lines

We inspected the prism and grating 1D and 2D spectra to identify any rest-frame ultraviolet (UV) emission lines above the level of the noise in both targets. We estimated emission line fluxes and equivalent widths from the continuum-subtracted spectra over five spectral channels (Extended Data Table 3). The uncertainties on line fluxes and equivalent width were determined by repeating the measurements on a sample of 2,000 spectra obtained by combining the spectra of the individual integrations with a bootstrap resampling technique.

**Extended Data Fig. 6:**
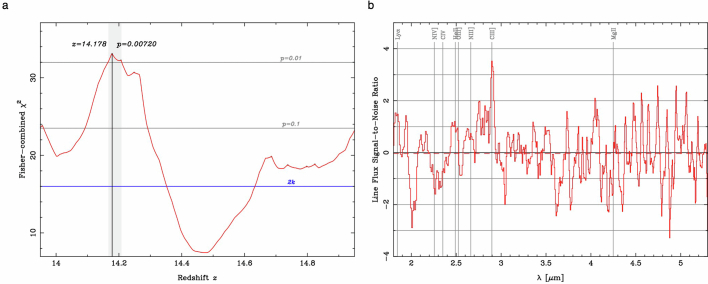
Emission line identification in the prism spectrum of JADES-GS-z14-0. **a**, Fisher-combined ${\chi }^{2}$ value as a function of redshift. The gray horizontal lines show Fisher-combined ${\chi }^{2}$ values at the combined p-values of 0.01 and 0.1. For reference, the blue line marked $2k$, where $k$ is the number of emission lines falling within the search window, reports the mean value of the ${\chi }^{2}$ statistic under the null hypothesis of no emission lines. There is a peak is at $z=14.178$ with a $p$-value of 0.00720. **b**, Potential line flux signal-to-noise ratios and the overlay shows the locations of the emission lines searched for at the peak of the combined $p$-value. The potential CIII]$\lambda 1909$ emission line has a level of significance of 3.6$\sigma$.

**Extended Data Fig. 7:**
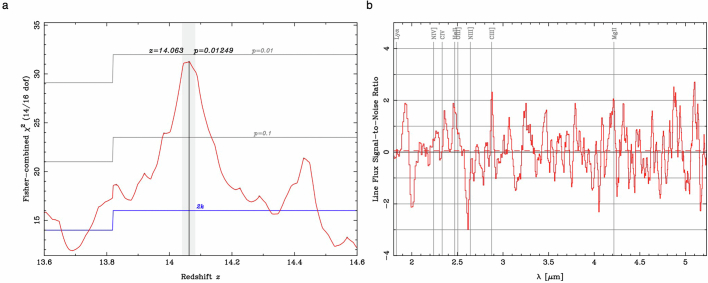
Emission line identification in the prism spectrum of JADES-GS-z14-1. **a**, Fisher-combined ${\chi }^{2}$ value as a function of redshift. The gray horizontal lines show Fisher-combined ${\chi }^{2}$ values at the combined p-values of 0.01 and 0.1. For reference, the blue line marked $2k$, where $k$ is the number of emission lines falling within the search window, reports the mean value of the ${\chi }^{2}$ statistic under the null hypothesis of no emission lines. There is a peak at $z=14.063$ with a $p$-value of 0.01249. **b**, Potential line flux signal-to-noise ratios and the overlay shows the locations of the emission lines searched for at the peak of the combined $p$-value. There are no emission lines with a level of significance higher than 3$\sigma$.

Given the uncertainties on the redshift based only on the Lyman break, we estimated the statistical significance of a set of emission lines (Extended Data Table 3) at different redshifts (Extended Data Figs. 6 and 7). In particular, we inferred the one-sided *P* value for each line at different redshifts. We then determined the combined *P* value of the set of lines by using Fisher’s method and used it to quantify the statistical significance of the spectroscopic redshifts (details in ref. ^3^).

In the prism spectrum for JADES-GS-z14-0, we identified only a potential CIII](*λλ*1907,1909) emission line at *z* = 14.178 with a level of significance of 3.6*σ* and the combined *P* value for the inferred redshift is 0.00720. The redshift is consistent within the error with that determined from the fitting of the Lyman-break profile, but follow-up observations will be required to confirm the emission line. We note that the presence of only carbon line in the rest-frame UV spectrum is consistent with other low-redshift studies concluding that CIII] might be the strongest rest-UV line after the Lyα line^66–69^.

Both the upper limits on the emission lines and the tentative detection of CIII] in JADES-GS-z14-0, with a rest-frame equivalent width of EW_0_ = 8.0 ± 2.3 Å (Extended Data Table 3), are consistent with those observed in lower-luminosity galaxies at *z* > 10 (ref. ^1,3^). On the other hand, if we compare JADES-GS-z14-0 with the most luminous galaxies at *z* > 10, such as GN-z11 (ref. ^44^) and GHZ2 (ref. ^16^), we would expect to detect both CIII] and CIV*λλ*1548,1551 in the prism spectra of our galaxies. This spectral difference may be due to an extremely low metallicity ($Z < 0.05\,{Z}_{\odot }$) or a large escape fraction of ionizing photons that reduces the emission by the gas in the interstellar medium^1,3^ or a different nature of the dominant ionizing flux^29^.

In the grating, we did not find any lines, and the 3*σ* upper limits, which were estimated by using the bootstrap resampling technique, are reported in Extended Data Table 3.

In both the prism and grating of JADES-GS-z14-1 we did not find any clear emission line feature with a level of significance higher than 3*σ*. We identified only the potential lines of CIII]*λ*1909 and MgII*λ*2795 at *z* = 14.063 with a signal-to-noise ratio of about 2. The combined *P* value for this redshift is 0.01249, suggesting that this solution is not statistically significant. We thus derived the upper limits on the emission lines and equivalent widths from both prism and grating spectra (Extended Data Table 3). The prism spectra also reveal an absorption feature at 2 μm but its significance is only 2*σ*. If it will be confirmed in future observations, it corresponds to CII*λ*1335 doublet absorption line.

### Possible large-scale structure association

**Extended Data Fig. 8:**
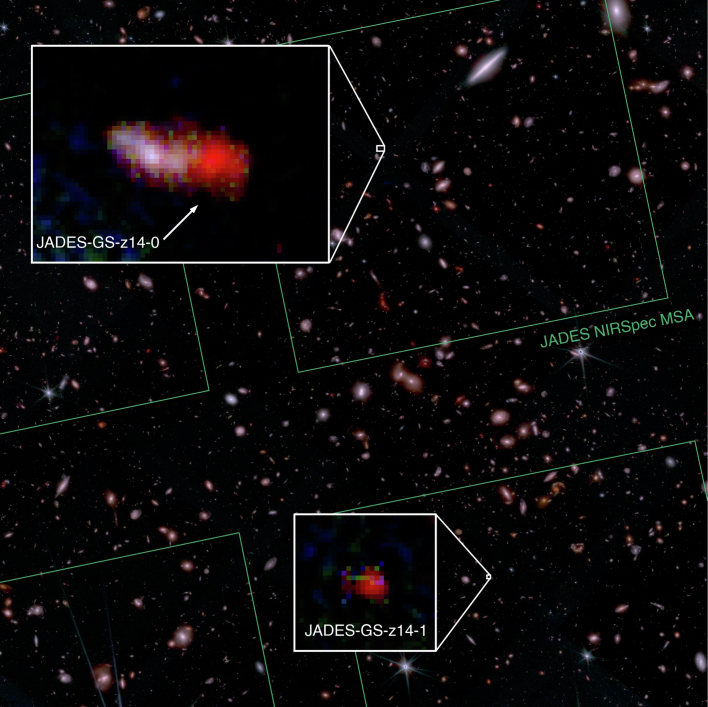
The sky around JADES-GS-z14-0 and JADES-z14-1. F444W/F200W/F090W false color red/green/blue image of the JADES imaging field^7^ (background) and F277W/F150W/F115W false color red/green/blue thumbnail images of the two $z \sim 14$ galaxies. The green outline illustrates the four quadrants of the NIRSpec micro-shutter array of the Cycle 1 NIRSpc 1287 program.

JADES-GS-z14-0 and JADES-GS-z14-1 are 1.9′ apart on the sky (Extended Data Fig. 8), which is 6.2 comoving Mpc at this redshift. The third candidate completes a roughly equilateral triangle, 1.7′ and 2.7′ away from the first two, respectively. These three galaxies form a mild angular over-abundance of the candidates from ref. ^12^, a fact that influenced the selection of this location for the programme 1287 deep NIRSpec pointing. The separation along the line of sight is imprecisely known, given the redshift uncertainties. If JADES-GS-z14-0 and JADES-GS-z14-1 are separated by 0.42 in redshift, then this would be about 60 comoving Mpc along the line of sight. However, the galaxies could be substantially closer, even potentially at the same redshift, if more unusual combinations of neutral hydrogen absorption and Lyα emission were present. Narrow-line redshifts will be needed to measure this. As galaxies at high redshift are expected to show a high clustering bias^70,71^, supported by numerous findings of inhomogeneity at *z* ≈ 7 (refs. ^72,73^), it seems likely that these galaxies are at least mildly associated in an extended large-scale structure.

### SED fitting

**Extended Data Fig. 9:**
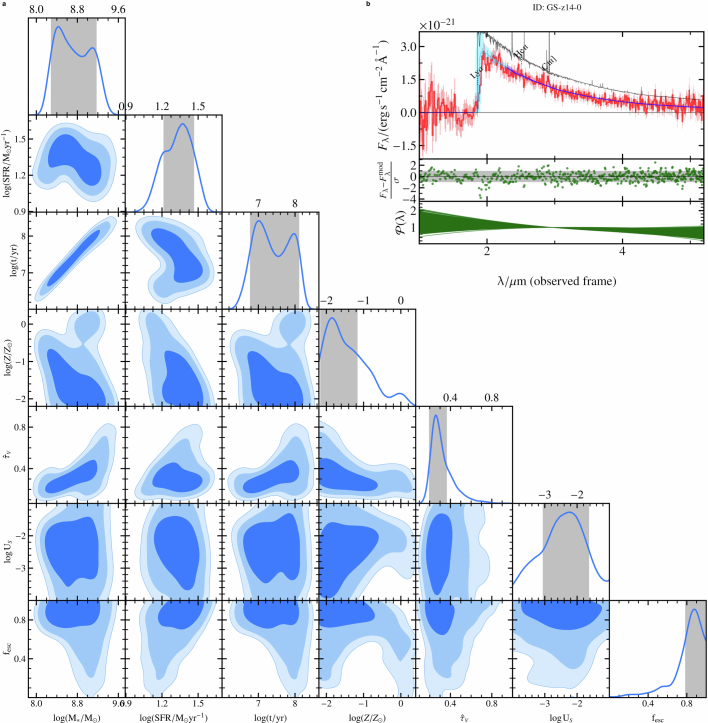
Beagle-based SED modeling of JADES-GS-z14-0. **a**, Posterior probability distributions obtained with Beagle for our fiducial fits, along with the observed spectrum and model prediction, for JADES-GS-z14-0. From left to right, the columns show the stellar mass ${M}_{* }$, star formation rate, age of the oldest stars $t$, (stellar and interstellar) metallicity $Z$, V-band attenuation optical depth ${\hat{\tau }}_{V}$, ionization parameter $\log {U}_{S}$, and escape fraction of ionizing photons ${f}_{{esc}}$. The 1D (marginal) posterior distribution of each parameter is plotted along the diagonal, where the shaded gray regions represent the 1$\sigma$ credible interval. The off-diagonal panels show the 2D (joint) posterior distributions, with the shaded blue regions representing the 1, 2, and 3$\sigma$ credible intervals. **b**, The observed spectrum (red line), model predictions (dark blue line), and model predictions before applying instrumental effects (i.e. line-spread function and calibration polynomial, grey line). The model predictions at $\lambda  < 1450$ Å are shown with a cyan line, to indicate that this region was masked during the fitting. In the central panel of the inset, we show the residuals in units of observed errors and the $\pm 1\sigma$ region in grey, while the bottom panel indicates the calibration polynomial.

**Extended Data Fig. 10:**
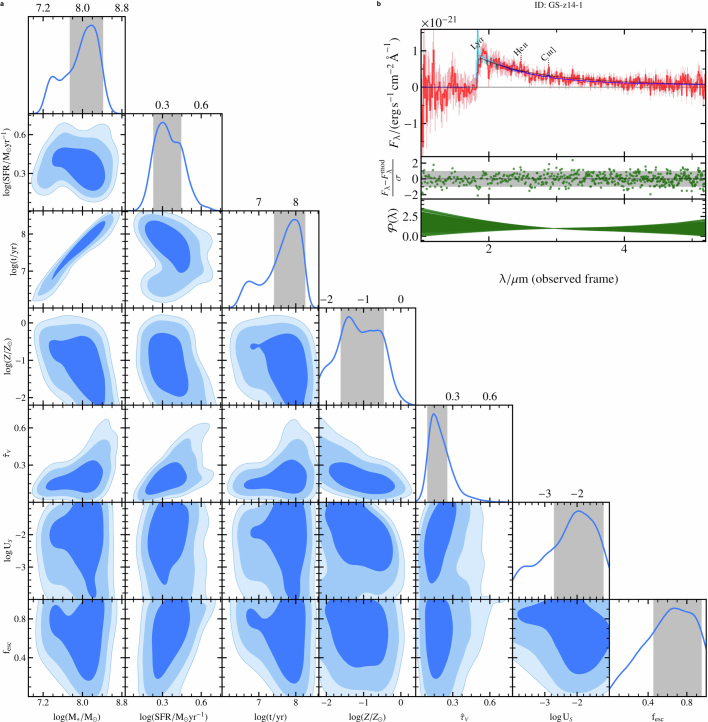
Beagle-based SED modeling of JADES-GS-z14-1. **a**, Posterior probability distributions obtained with Beagle for our fiducial fits, along with the observed spectrum and model prediction, for JADES-GS-z14-1. From left to right, the columns show the stellar mass ${M}_{* }$, star formation rate, age of the oldest stars $t$, (stellar and interstellar) metallicity $Z$, V-band attenuation optical depth ${\hat{\tau }}_{V}$, ionization parameter $\log {U}_{S}$, and escape fraction of ionizing photons ${f}_{{esc}}$. The 1D (marginal) posterior distribution of each parameter is plotted along the diagonal, where the shaded gray regions represent the 1$\sigma$ credible interval. The off-diagonal panels show the 2D (joint) posterior distributions, with the shaded blue regions representing the 1, 2, and 3$\sigma$ credible intervals. **b**, The observed spectrum (red line), model predictions (dark blue line), and model predictions before applying instrumental effects (i.e. line-spread function and calibration polynomial, grey line). The model predictions at $\lambda  < 1450$ Å are shown with a cyan line, to indicate that this region was masked during the fitting. In the central panel of the inset, we show the residuals in units of observed errors and the $\pm 1\sigma$ region in grey, while the bottom panel indicates the calibration polynomial.

We fit JADES-GS-z14-0 (Extended Data Fig. 9) and JADES-GS-z14-1 (Extended Data Fig. 10) following a similar approach to that of Hainline et al.^3^ and Curtis-Lake et al.^1^. In summary, we use the BEAGLE tool (Bayesian analysis of galaxy SEDs)^74^ to fit the combined R100 NIRSpec spectra and NIRCam + MIRI photometry. We fit the entire spectrum uncorrected for slit losses and mask the rest-frame region 1,150–1,450 Å to avoid biases arising from the Lyα damping wing, which we do not model in BEAGLE. We fit the NIRCam wide-bands F090W, F115W, F150W, F200W, F277W, F356W, F444W and the MIRI F770W band, using the values and errors reported in Extended Data Table 2. We adopt Gaussian priors for the redshift of the sources, centred on *z* = 14.32 and with *σ*_z_ = 0.2 for JADES-GS-z14-0, and centred on *z* = 13.9 and with *σ*_z_ = 0.17 for JADES-GS-z14-1. To account for the wavelength-dependent slit losses of NIRSpec, which are especially important for the extended object JADES-GS-z14-0, we include in the fitting a second-order polynomial, which is applied to the model spectrum before comparing it with the observed one. This enables the model to reproduce consistently the NIRCam + MIRI photometry and uncorrected NIRSpec spectrum. The inset in Extended Data Fig. 9, and in particular the difference between the grey (model before the polynomial correction) and blue lines (model with the applied correction), shows the importance of this correction for JADES-GS-z14-0. We do not use the first-order polynomial correction estimated in the section ‘NIRSpec observations and data reductions’ because we want to take into account the uncertainties associated with slit losses correction directly in the Bayesian SED fitting.

As in ref. ^3^, we use an updated version of the Bruzual and Charlot^75^ stellar population synthesis models (see ref. ^76^ for details), along with the photoionization models of Gutkin et al.^77^. We assume a Chabrier^78^ initial mass function with lower and upper mass limits of 0.1 *M*_⊙_ and 300 *M*_⊙_, respectively. We fit three different models, in which we vary assumptions on the star-formation history and escape fraction of ionizing photons. Similar to ref. ^3^, we find that the blue UV slopes of our objects and the absence of (securely) detected emission lines, can be explained by (1) a SFR that suddenly drops to very low values during the last 10 Myr of star formation, but with most stars being a few tens of million years old, as they can produce blue UV slopes. This scenario indicates an unlikely fine-tuning of the star-formation history of these galaxies. (2) A metal-enriched gas (near-to-Solar metallicity), as such a large metallicity would also suppress the high-ionization UV lines (but also produce redder UV slopes). (3) A large escape fraction of ionizing photons. This last model is our fiducial one, because, as we extensively discussed in ref. ^3^, it is the only model that can simultaneously reproduce the observed blue UV slopes and the absence of UV emission lines.

Our fiducial model is thus described by seven adjustable parameters: the total stellar mass formed *M*_tot_, age of the oldest stars *t*, stellar metallicity ${Z}_{* }$, gas ionization parameter log*U*_s_, V-band dust attenuation optical depth ${\hat{\tau }}_{V}$ (that is, *A*_V_/1.086), escape fraction of ionizing photons *f*_esc_ and redshift *z*. We assume the ref. ^79^ two-component dust attenuation model, fixing the fraction of attenuation arising from stellar birth clouds to *μ* = 0.4. We further fix the metal’s depletion factor to $\xi =0.1$. Note that in our model the stellar metallicity ${Z}_{* }$ and interstellar metallicity *Z*_ism_ are equal, whereas the gas abundance of a metal further depends on the metal depletion.

From BEAGLE-based SED modelling we find that the JADES-GS-z14-0, once corrected for the gravitational lensing amplification, has a stellar mass of ${\log }_{10}\left({M}_{{\rm{star}}}/{M}_{\odot }\right)={8.6}_{-0.2}^{+0.7}$ and a SFR, averaged over the last 10 Myr, of ${{\rm{SFR}}}_{10}=19\pm 6\,{M}_{\odot }{{\rm{yr}}}^{-1}$, resulting in a specific SFR (sSFR) of sSFR = SFR/*M*_star_ = 45 Gyr^−1^. We estimate a gas-phase metallicity of $\log \left(Z/{Z}_{\odot }\right)=-{1.5}_{-0.4}^{+0.7}$ and a dust attenuation of *A*_V_ ≈ 0.3 mag assuming a standard nebular continuum powered by OB stars. JADES-GS-z14-1 is less massive, with ${\log }_{10}\left({M}_{{\rm{star}}}/{M}_{\odot }\right)={8.0}_{-0.3}^{+0.4}$ and a SFR of ${{\rm{SFR}}}_{10}={2.0}_{-0.4}^{+0.7}\,{M}_{\odot }{{\rm{yr}}}^{-1}$. We infer a sSFR of about 18 Gyr^−1^. For this galaxy, we estimate a metallicity of $\log \left(Z/{Z}_{\odot }\right)=-{1.1}_{-0.6}^{+0.6}$ and a dust attenuation of *A*_V_ ≈ 0.2 mag. We remind that the errors quoted here refer only to the internal statistical errors of our model with the above assumptions. Notably, the inference of stellar mass is known to be sensitive to assumptions, with variations of about 0.2 dex depending on the SED fitting code and star-formation histories allowed in the model^28^. More exotic deviations in astrophysics, such as variations in the stellar initial mass function^80^, could create further differences.

### Morphological analysis

To determine the extension of JADES-GS-z14-0 and JADES-GS-z14-1, we initially extracted the radial surface brightness profile from the NIRCam F200W images, which are a compromise between signal to noise and angular resolution for these galaxies.

We have determined the average surface brightness from concentric radial annuli centred at the position of the target and radius width of 0.03″. For JADES-GS-z14-0 we have masked the image at right ascension coordinate greater than 5.30829890° (that is, 0.1″ from the galaxy centre) to remove the contamination from the foreground source. Figure 3 shows the normalized surface brightness profile for both targets. The emission of JADES-GS-z14-0 is more extended than the light profile of the star (that is, point-like source) observed in the NIRCam field. JADES-GS-z14-0 is also more extended than the surface brightness profile of GN-z11 (ref. ^46^) and GHZ2 (ref. ^16^). On the other hand, JADES-GS-z14-1 seems more compact and consistent with a point-like source.

We have also modelled the morphology of the galaxies with ForcePho, which enables us to fit the light distribution of individual exposures across all filters simultaneously while taking into account the substantial change in the NIRCam point spread function with wavelength. In this case we do not need to apply any mask in the NIRCadm image as ForcePho models simultaneously the target and the neighbouring galaxies returning the morphological and photometric parameters for all sources in the field.

In the F162M image, JADES-GS-z14-0 is near to the edge of or entirely missed by several of the individual exposures from the 3215 programme^50^. By modelling the individual exposures simultaneously ForcePho avoids the correlated noise caused by mosaicing, which can be particularly difficult to quantify when the source is near a large gradient in exposure time such as JADES-GS-z14-0. We have adopted a single Sérsic profile for the *z* ≈ 14 galaxies and for the foreground sources within 5 arcsec from the targets. ForcePho fits these profiles simultaneously and with full posterior sampling, allowing us to measure the uncertainties in the profile and the covariance of the fluxes between sources that seem blended, such as JADES-GS-z14-0 and its low-redshift neighbour.

**Extended Data Fig. 11:**
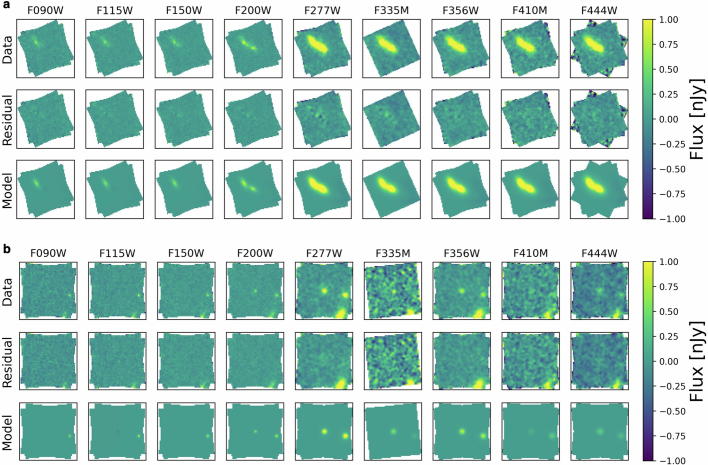
ForcePho model fitting. **a–b**, The data, residual, model, and fluxes with the recovered galaxy component for JADES-GS-z14-0 (**a**) and JADES-GS-z14-1 (**b**) in the multiple NIRCam bands. Each frame shows a ${2}^{{\prime\prime} }\times {2}^{{\prime\prime} }$ stamp centred on the location of each galaxy. The figure shows that the model has fit the data well within all bands without leaving significant residuals.

Data, residuals and the model are shown in Extended Data Fig. 11. A single component is sufficient to match the data of both JADES-GS-z14-0 and JADES-GS-z14-1. In both cases, the surface brightness profile is consistent with an Sérsic profile with an index of around one. For the brightest of the two galaxies, we have determined a deconvolved half-light radius of 260 pc, whereas the compact size of JADES-GS-z14-1 has returned just an upper limit of 160 pc.

### Dust enrichment

**Extended Data Fig. 12:**
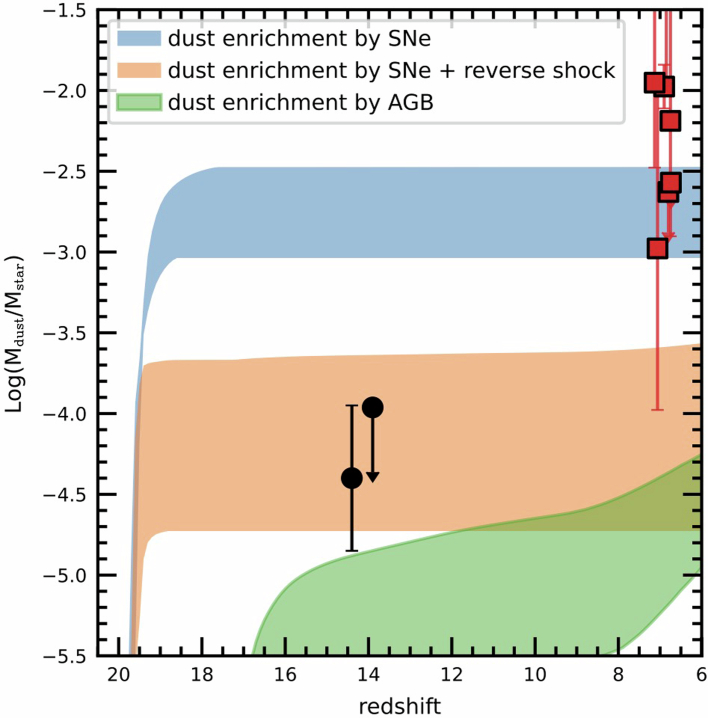
Dust to stellar mass ratio. Dust enrichment models by supernovae (SNe) with no (blue) and with (orange) the effect of the partial dust destruction due to the reverse shock, and asymptotic giant branch (AGB) stars from a single star-formation burst at $z=20$. The relative contribution of the SNe and AGB dust formation are estimated assuming an initial progenitor metallicity in the range $0.01 < Z/{Z}_{\odot } < 0.1$^46^. Black circles illustrate the dust-to-stellar mass ratio of JADES-GS-z14-0 and JADES-GS-z14-1. A proxy of the dust masses of the two $z \sim 14$ galaxies has been estimated from the ${A}_{V}$ parameter, assuming that dust is distributed in a sphere with a radius as large as the galaxy radius^34^. We also report in red squares the dust-to-stellar mass ratio of HST-selected, UV-bright galaxies at $z \sim 6\mbox{--}7$, with dust measurements from ground-based millimeter telescopes^37,59,83^.

Following ref. ^34^ and assuming the prescription of ref. ^81^ for the dust attenuation law, *A*_V_ could be used as a proxy for the dust mass: ${M}_{{\rm{d}}}\approx 2.2\times {10}^{4}{A}_{{\rm{V}}}{\left({r}_{{\rm{d}}}/100{\rm{pc}}\right)}^{2}{M}_{\odot }$, where *r*_d_ is the radius over which the interstellar medium dust is assumed to extend. However, we stress that the *A*_V_ parameter is not an accurate measure of the actual amount of dust as this parameter is simply estimated from the spectral fitting assuming an dust attenuation law. Assuming that the spatial extension of the dust is as large as the size of the galaxy (that is, *r*_d_ = *r*_UV_), we derived a dust mass ${M}_{{\rm{d}}}=5\times {10}^{4}{M}_{\odot }$ and ${M}_{{\rm{d}}} < 0.5\times {10}^{4}{M}_{\odot }$ for the two galaxies, respectively. Comparing these masses with the stellar masses, we find a dust-to-stellar mass fraction of less than 10^−4^ for both galaxies (Extended Data Fig. 12), which is a factor greater than ten lower than those inferred in galaxies at $z\approx 6\mbox{--}7({10}^{-2}\mbox{--}{10}^{-3})$ (refs. ^37,82,83^) and that predicted by supernova models without reverse shock^36,84^. Extended Data Fig. 12 shows the timescales to reach the asymptotic dust-to-stellar mass ratio due to various dust enrichment processes and assuming that galaxies formed at *z* = 20 from a single star-formation burst (review by Schneider and Maiolino^36^). In the first few million years, the dust is dominated by supernovae, and in less than 5 Myr, the galaxy has already reached a dust-to-stellar mass ratio of around 1/1,000. Even assuming a different realistic star-formation history, the dust-to-stellar mass reaches the asymptotic values of less than 10 Myr. Reverse shocks created by the interaction between the expanding supernova blast wave and the interstellar medium can limit the effective dust enrichment by supernovae^85–88^, as indicated by the orange shaded region in Extended Data Fig. 12. However, the efficiency of dust destruction due to reverse shocks is still debated, and different models predict different survival rates^36^.

## Online content

Any methods, additional references, Nature Portfolio reporting summaries, source data, extended data, supplementary information, acknowledgements, peer review information; details of author contributions and competing interests; and statements of data and code availability are available at 10.1038/s41586-024-07860-9.

## Figures and Tables

**Extended Data Table 1:**
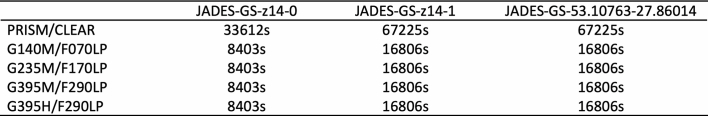
Exposure times

The total on-source exposure time in seconds for each NIRSpec disperser-filter combinations used in this work.

**Extended Data Table 2:**
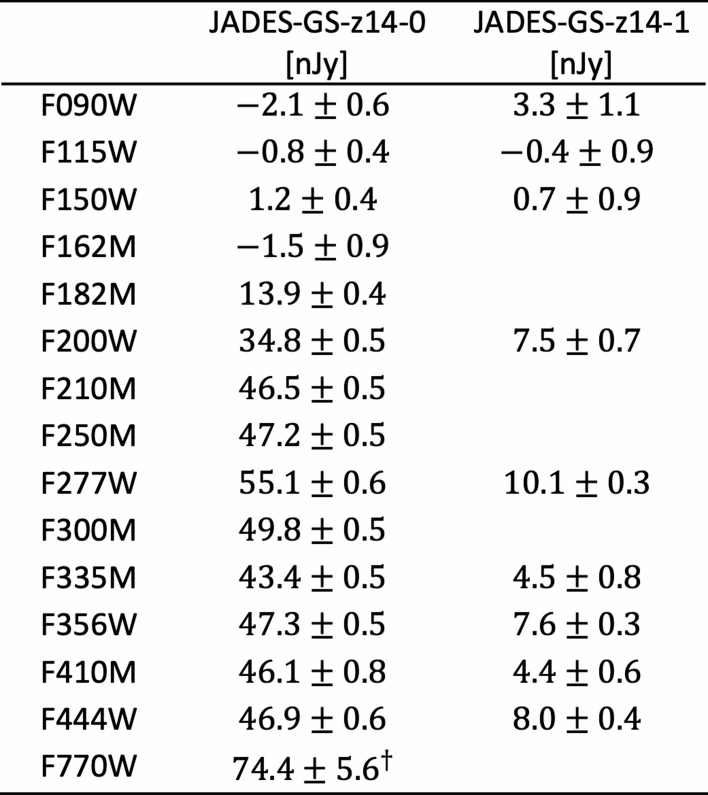
NIRCam and MIRI fluxes of JADES-GS-z14-0 and JADES-GS-z14-1

ForcePho photometry of JADES-GS-z14-0 and 0.2″ diameter circular aperture photometry of JADES-GS-z14-1. ^†^ from Helton et al.^28^.

**Extended Data Table 3:**
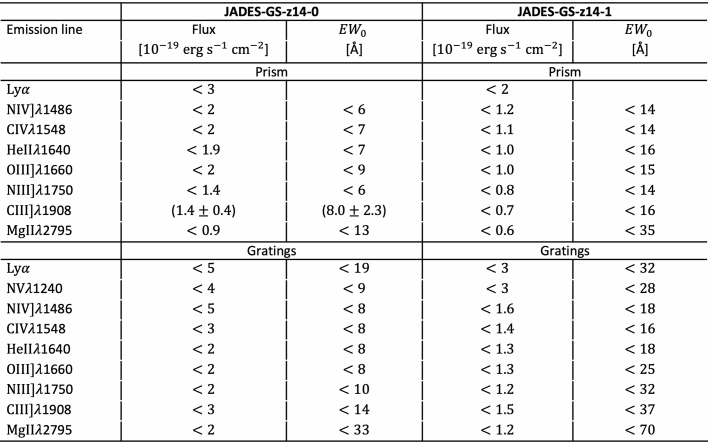
Spectral measurements from prism and gratings spectra

Emission line fluxes and rest-frame equivalent widths measured in both prism and gratings spectra. Fluxes are in units of 10^−19^ erg s ^−1^ cm^−2^, rest-frame equivalent widths ($E{W}_{0}$) are in (rest-frame) Å. We adopted 3$\sigma$ upper limits and emission line fluxes have been estimated over a resolution element of 5 spectral channels of R1000 data.

## Data Availability

The NIRCam data that support the findings of this study are publicly available at https://archive.stsci.edu/hlsp/jades. The reduced spectra that support the findings of this study are publicly available at Zenodo (10.5281/zenodo.12578542) (ref. ^89^).

## Code availability

The AstroPy^90,91^ software suite is publicly available, as ForcePho. BEAGLE is available by means of a Docker image upon request at http://www.iap.fr/beagle/.
